# Physical fitness as a predictor of herniated lumbar disc disease – a 33-year follow-up in the Copenhagen male study

**DOI:** 10.1186/1471-2474-14-86

**Published:** 2013-03-09

**Authors:** Marie B Jørgensen, Andreas Holtermann, Finn Gyntelberg, Poul Suadicani

**Affiliations:** 1 The National Research Centre for the Working Environment, Lersø Parkallé 105, Copenhagen, DK-2100, Denmark; 2 The Copenhagen Male Study, Epidemiological Research Unit, Department of Occupational and Environmental Medicine, Bispebjerg University Hospital, Copenhagen, Denmark

**Keywords:** Lumbar disc herniation, Prospective cohort, Physical workload, Physical fitness, Height

## Abstract

**Background:**

The role of physical fitness (VO_2_Max (mlO_2_*min^-1^*kg^-1^)) as a risk factor for herniated lumbar disc disease (HLDD) is unknown. The objective of this study was to examine the association between aerobic (physical) fitness and risk of hospitalisation due to HLDD in a long-term follow up.

**Methods:**

The Copenhagen Male Study is a prospective cohort study established in 1970–71. At baseline, 5,249 men answered a questionnaire about their history of back disease, physical and psychosocial working conditions, lifestyle and social class. Height and weight was measured and aerobic capacity (physical fitness) was estimated based on a submaximal bicycle test. Information about hospitalization due to HLDD was obtained from the National Hospital Register covering the period 1977 – 2003. Hazard Ratios (HR) were calculated by Cox’s proportional hazard regression model.

**Results:**

Among 3,833 men without history of low back disorders, 64 were hospitalized due to HLDD. The cumulative incidence of HLDD was 1.7% (n=34) among men with low physical fitness (15–32 ml O_2_*min^-1^*kg^-1^), and 1.7% (n=30) among men with high physical fitness (33–78 ml O_2_*min^-1^*kg^-1^). In a final model, adjusted for relevant confounders, the HR (95% CI) for HLDD for those with high physical fitness was 0.88 (0.51-1.50) compared to those with low physical fitness. In the same model, HR for men often exposed to strenuous work compared to those seldom or never exposed to strenuous work was 3.91(1.82-8.38). Also body height was a significant predictor.

**Conclusions:**

Physical fitness is not associated with hospitalisation due to HLDD, and the only modifiable risk factor for hospitalisation due to HLDD seems to be strenuousness at work.

## Background

Low back disorders are frequent in the western societies with considerable individual, societal and economical consequences [1]. Investigation of modifiable risk factors for development of low back pain has therefore been requested [1].

Aerobic capacity (physical fitness) is a highly modifiable factor depending the on level of physical activity [2], and low physical fitness has previously been shown to be associated with increased risk of low back disorders [3-5]. Physical activity is among the most common recommendations for prevention and rehabilitation of low back disorders [1], and recently in a 5-year follow-up of 41 individuals Elfering *et al.*[6] found a higher risk of lumbar disc degeneration among those who did not participate in sports activities. A recent cross-sectional study of police workers by Heneweer *et al.*[5] found that individuals with a high physical fitness had a lower prevalence of low back pain than others. In the same study a U-shaped association was found between physical activity at work and in leisure time and low back pain. The authors concluded that physical fitness, rather than self-reported physical activities, is more strongly associated with low back pain. However, the physiological mechanism for the association between low aerobic capacity and elevated risk of low back disorders is questionable and needs further investigation with objective data. Potentially, the difference is merely an effect of differences in lifestyle.

Although herniated lumber disc disease (HLDD) is only the cause of low back pain in a small number of individuals with low back pain, in contrast to the unspecific nature of low back pain generally, it is a well-defined pathologic condition. Also, HLDD should be considered a serious manifestation of low back disorder, making it relevant to study if low physical fitness may be a risk factor for developing HLDD. No previous studies have addressed this potentially important issue. A previous paper from the Copenhagen Male Study showed that frequent exposure to strenuous physical activity at work was a strong risk factor for later hospitalisation due to HLDD. In that paper, the potential role of physical fitness was not considered [7].

In the present study we tested the hypothesis that low physical fitness (objectively measured estimation of aerobic capacity) is a risk factor for hospitalisation due to HLDD.

## Methods

### Study population

The Copenhagen Male Study was established in 1970–1971 as a prospective cohort study of physical fitness and cardio-vascular disease in employees in 14 private and public companies in Copenhagen. All men aged 40–59 years were invited; 5,249 men, 87% of potential participants, took part in the examinations at baseline [8,9]. In the present study, only men without a history of back disorders were included.

Baseline data were obtained by interview based on a precedent structured questionnaire, and a clinical examination including measurement of height and weight [10], and indirect measurement of aerobic capacity (physical fitness) was performed with a bicycle ergometer [11]. Thirty-five men with orthopaedic problems unable to perform the bicycle test were excluded from the study. Information on working conditions, lifestyle, and general health was obtained from the questionnaire. Variables that could represent potential risk factors for lumbar intervertebral disc herniation were chosen [12-17]. Details on the questionnaire have previously been published [10].

Physical fitness Heart rate was measured during submaximal bicycle work in steady state with the aid of a stopwatch and stethoscope. The loads used were 100, 150 and 200 W. One, two or in a few cases three different loads were used. The loads chosen in each case were determined from the weight and age of the person or heart rate during the first minute of the test, and the estimation of VO_2_Max was accomplished with the aid of Åstrand’s nomogram [18]. The correlation between directly and indirectly measured physical fitness is high. The method used has previously been described in detail [8].

Physical occupational workload was assessed from one question addressing ergonomic load to the back: “Is your work a) sedentary, b) slightly physical without lifting, c) physical with some lifting, or d) hard physical with heavy lifting, shovelling or the like”? The two first categories were condensed in the analyses. Additionally, a more general question about physical workload was asked: “Do you perform strenuous work (regularly resulting in sweating)?” Answer options were: “often”, “occasionally” and “seldom or never”.

Physical activity in leisure time was classified into one of four different groups: Predominantly sedentary, slightly active, fairly active or very active. For analytical purposes answers in the two middle groups were condensed.

Mental stress at work and during leisure time was evaluated separately by asking: “Do you feel under mental stress when performing your job/during leisure time?” Answer options were: “Seldom” and “Regularly”. In addition it was asked whether sedatives were taken regularly, occasionally or never.

Lifestyle factors included current and previous tobacco and alcohol consumption.

Health related factors included questions on present or previous episodes of back pain or back injury. Answer options were yes and no.

Anthropometrics included height and weight based on measurements and body mass index (BMI) calculated as kg * m^–2^. In separate analyses of height and weight the study population was divided into thirds based on tertile values. BMI was divided into three groups: Normal weight or lean: BMI < 25, overweight: BMI 25–29.9, and obese: ≥ 30.

Social class was grouped in five categories based on educational level, job type and position in terms of number of subordinates [8,19]. Typical jobs in the cohort were as follows. Social class I: head of department, officer, civil engineer. Social class II: head clerk, engineer, resident architect. Social class III: engine driver, train guard. Social class IV: skilled workers. Social class V: unskilled worker, driver.

End-point was hospitalization due to herniated lumbar disc disease identified in the National Hospital Register between 1977 and 2003. Code 725.11 from the International Classification of Diseases (ICD), 8^th^ Revision was applied from 1977 to 1994, and code M51.1 from ICD 10^th^ Revision from 1994 to 2003.

### Statistical analyses

The analyses were performed using SPSS for Windows [20,21]. Relative risks were estimated by exp(β), where β is the hazard coefficient for the variable of interest in a Cox’s proportional hazards regression model [22] with the maximum likelihood ratio method and the use of a backward stepwise elimination procedure in analyses adjusted for age only, and in a final model including also other potentially relevant variables. Assumptions for use of Cox’s proportional hazards were met by inspection of the log minus log function at the covariate mean. A two-sided probability value of p ≤ 0.05 was *a priori* taken as significant.

### Ethics

The ethics committee for medical research in the County of Copenhagen approved the study in connection with a follow-up of the cohort in 1985–86; in 1970 no committee had been established.

## Results

At baseline, 1,412 of the men (26.9%) reported a history of back disorder. The 3,833 individuals without such a history were entered in the study. A number of 108 men were hospitalized due to herniated lumbar disc disease during the study period 1977 to 2003; of those, 64 men without previous history of low back pain or injury were hospitalized – the group analysed in the present study.

Table 1 shows potential predictors of hospitalisation for *Herniated lumbar disc disease* among the 3,833 men *without* history of any back disease at baseline in 1970–71. In analyses adjusted for age only, significant associations were found for occasional as well frequent exposure to strenuous work, and for increasing height and weight. Physical fitness was not significantly associated with HLDD.

**Table 1:** **Potential predictors of hospitalisation for *Herniated lumbar disc disease *during the period 1977–2003 among 3,833 men *without *history of any back disease at baseline in 1970–71**

	**Herniated lumbar disc disease, n (%)**	**Age-adjusted HR (95% CI)**
Physical fitness (VO_2_Max)		
Low, - 32 (n= 2055)	34 (1.7%)	1 (reference)
High, 33 + (n= 1778)	30 (1.7%)	0.85 (0.51-1.40)
Ergonomic load to the back		
Low (n=3,073)	47 (1.5%)	1 (reference)
Medium (n=582)	13 (2.2%)	1.54 (0.83-2.84)
High (n = 79)	3 (3.8%)	2.80 (0.87-9.0)
**Strenuous work**		
** Seldom/never (n = 2,328)**	**26 (1.1%)**	**1 (reference)**
** Occasionally (n= 1,186)**	**26 (2.2%)**	**2.09 (1.21-3.61)**
** Often (n= 247)**	**10 (4.0%)**	**3.95 (1.90-8.20)**
Leisure time physical activity		
Low (n= 647)	13 (2.0%)	1 (reference)
Medium (n = 2,710)	43 (1.6%)	0.72 (0.38-1.33)
High (n = 365)	7 (1.9%)	0.78 (0.31-1.97)
Social class		
I (n = 676)	8 (1.2%)	1 (reference)
II (n = 395)	5 (1.3%)	1.06 (0.34-3.24)
III (n = 717)	13 (1.8%)	1.58 (0.65-3.82)
IV (n = 1,542)	28 (1.8%)	1.57 (0.71-3.46)
V (n = 497)	10 (2.0%)	2.22 (0.87-5.65)
**Height, cm**		
** - 171 (n = 1,283)**	**12 (0.9%)**	**1 (reference)**
** 172-177 (n = 1,355)**	**29 (2.1%)**	**2.06 (1.05-4.05)**
** 178 + (n = 1,186)**	**21 (1.8%)**	**1.60 (0.78-3.26)**
**Weight, kg**		
** - 72 (n = 1,282)**	**14 (1.1%)**	**1 (reference)**
** 73-80 (n = 1,278)**	**21 (1.6%)**	**1.51 (0.77-2.98)**
** 81 + (n = 1,271)**	**29 (2.3%)**	**2.15 (1.13-4.07)**
BMI, kg*m ^–2^		
- 25 (n = 1,858)	28 (1.5%)	1 (reference)
> 25–29.9 (n = 1,712)	31 (1.8%)	1.30 (0.78-2.17)
30 + (n = 250)	3 (1.2%)	0.95 (0.28-3.12)
Under mental stress at work		
Seldom (n = 3,002)	54 (1.8%)	1 (reference)
Regularly (n = 821)	10 (1.2%)	0.64 (0.32-1.26)
Under mental stress in leisure time		
Seldom (n = 3,574)	62 (1.7%)	1 (reference)
Regularly (n= 249)	2 (0.8%)	0.47 (0.11-1.94)
Use of sedatives		
Never (n = 2,998)	51 (1.7%)	1 (reference)
Regularly/occasionally (n=827)	13 (1.6%)	1.08 (0.58-2.00)
Smoking		
Current (n = 2798)	41 (1.5%)	1 (reference)
Previously (n = 705)	17 (2.4%)	1.43 (0.56-3.63)
Never (n = 328)	6 (1.8%)	0.95 (0.40-2.23)
Daily alcohol use, beverages		
0 (n = 1,271)	21 (1.7%)	1 (reference)
1-2 (n = 1,815)	32 (1.8%)	1.08 (0.62-1.87)
3-5 (n = 611)	9 (1.5%)	1.03 (0.47-2-26)
>= 6 (n = 123)	2 (1.6%)	1.22 (0.28-5.21)

Adjusted for age only. Significant associations (p < 0.05) are highlighted (bold). HR = hazard ratio; CI = confidence interval.

Table 2 shows characteristics of men with low and high physical fitness (cut-off at median=32) among men without history of low back pain/injury. Included in the table are only factors significantly associated with HLDD risks in Table 1. Men with low physical fitness were slightly older, and they weighed more. These two factors could thus be regarded as conventional potential confounders and were included in the final model shown in Table 3.

**Table 2:** Characteristics of men with low and high physical fitness (cut-off at median=32) among men without history of low back pain/injury

	**Level of physical fitness**		
	**Up to median**	**Above median**	**p**^**a**^
	**VO**_**2**_**MAX range: 15-32**	**VO**_**2**_**MAX range: 33-78**	
	**n=2055**	**n=1778**	
Physical fitness			
VO_2_MAX (mlO_2_/kg/min)	27.5 (3.5)	38.8 (5.4)	-
Strenuous work, %			
Seldom/never	62.4	61.3	
Occasionally	31.0	32.2	0.72
Often	6.6	6.5	
Height, %			
- 171 cm	33.5	33.6	
172-177 cm	35.8	35.0	0.83
178 + cm	30.7	31.4	
Height, mean (SD)	174.3 (6.5)	174.3 (6.5)	0.87
**Weight, %**			
**- 72 kg**	**30.3**	**49.5**	
** 73-80 kg**	**30.7**	**30.7**	**<0.001**
** 81 + kg**	**39.0**	**19.9**	
**Weight, mean (SD)**	**79.2 (10.4)**	**74.1 (9.1)**	**<0.001**
**Age, years**	**49.8 (5.4)**	**47.4 (5.1)**	**<0.001**

Included in the table are only factors significantly associated with HLDD risk in Table 1. Values presented are mean (SD) or frequency in per cent. Significant associations (p < 0.05) are highlighted (bold). ^a^: p-values of unpaired t test or Chi-square test (likelihood ratio) where appropriate.

**Table 3:** Predictors of hospitalisation due to HLDD including all factors from Table 2 including physical fitness

**Covariate**	**HR (95% CI)**
**Strenuous work (work resulting in sweating)**	
** Seldom/never (n = 2,328)**	**1 (reference)**
** Occasionally (n= 1,186)**	**2.37 (1.36-4.13)**
** Often (n= 247)**	**3.91 (1.82-8.38)**
**Height, cm**	
** - 171 (n = 1,283)**	**1 (reference)**
** 172-177 (n = 1,355)**	**2.18 (1.10-4.32)**
** 178 + (n = 1,186)**	**1.88 (0.92-3.86)**
Not in the final model, p > 0.10:	
Physical fitness (VO_2_Max)^a^	
Low, - 32 (n= 2055)	1 (reference)
High, 33 + (n= 1778)	0.88 (0.51-1.50)
Weight, kg^a^	
- 72 (n = 1,282)	1 (reference)
73-80 (n = 1,278)	1.40 (0.69-2.86)
81 + (n = 1,271)	1.81 (0.89-3.66)

Cox proportional hazards regression using backward elimination. Variables are ranked after statistical strength of association with the outcome following adjustment. Significant associations (p < 0.05) are highlighted (bold). ^a^: association with herniated lumbar disc disease at time of withdrawal from the model.HR = hazard ratio; CI = confidence interval.

Table 3 shows the result of the Cox proportional hazards regression analysis using backward elimination. In addition to physical fitness, age and weight, we included in the analysis the two factors previously shown to be risk factors for HLDD in the Copenhagen Male Study. In the fully adjusted model, only strenuous work (HR (95% CI) 2.37 (1.36-4.13) and 3.91 (1.82-8.38) for occasionally and often, respectively) and body height (HR (95% CI) 2.18 (1.10-4.32) and 1.88 (0.92-3.86) for 172-177 cm and 178+ cm, respectively) remained significantly associated with HLDD. Men with high physical fitness had a slightly lower risk for HLDD, far from reaching statistical significance, (HR = 0.88 and 95% CI = 0.51-1.50).

## Discussion

This study is the first to investigate the relation between objectively measured physical fitness and hospitalization due to HLDD in a prospective design. The hypothesis that low physical fitness is a risk factor for hospitalization due to HLDD was not supported.

As mentioned in the introduction, a beneficial effect on low back disorders should be expected with increasing leisure time physical activity (LTPA) sufficiently intense to increase cardio-respiratory fitness. However, in this study, neither self-reported LTPA nor the objectively measured physical fitness was predictive of later hospitalization due to HLDD. From a theoretical point of view, physical activity should increase the resistance of the intervertebral discs as discussed by Nachemson [19]. So, it cannot be ruled out that the negative finding in our study might be caused by a lack of statistical power (a type 2 error). Still, not even a tendency of an association was observed with an incidence of HLDD of 1.7% among men with a rather low physical fitness (mean (SD) 27.5 (3.5)), and 1.7% among men with a quite high physical fitness level (mean (SD) 38.8 (5.4)). Also, age-adjusted mean baseline values of physical fitness were practically identical for those who were later hospitalized due to HLDD and those who were not, 32.74 and 32.75, respectively (not shown in table). Furthermore, to gain an even larger exposure contrast between men with a high and a low physical fitness, we compared the incidence of HLDD between the lowest and the highest quintiles of physical fitness (not shown in table). The incidence among men with a fitness level of 15–26 (lowest quintile) was 1.9%; among men in the highest quintile 39–78 (highest quintile) it was 2.1%. Based on the above reasoning and the results, we find it unlikely that a lack of statistical power explains our findings.

Since no previous studies have investigated the association between physical fitness and HLDD, we are not able to compare our results with those of others. Previous studies on low back pain may indicate that a high level of physical fitness may be associated with less LBP as demonstrated by Heneweer et al. [5] in a cross-sectional case–control study. However, as mentioned, only a small fraction of people who suffer from low back pain do so due to HLDD [20]. Therefore, it is plausible that high physical fitness may prevent low back pain, but not HLDD.

As previously shown and indicated in Table 3 in this paper, strenuous occupational work is a significant predictor for risk of hospitalization due to HLDD. It is well known that strenuousness at work is a combined factor of both the external physical work demand and the physical fitness of the worker [21]. However, strenuous work tasks in this study include lifting of heavy burdens and awkward body positions, which may rather call for high muscular capacity than aerobic capacity. Occupational exposure to strenuous work tasks may not necessarily induce a training effect in muscles and connective tissues in the low back region. This is supported by a former study among elite athletes [22]. In contrast, strenuous exposures may be harmful because it may overload tissues of the lower back [19]. Instead, physical training during leisure has shown to improve capacity and reduce risk of low back disorders [23], but Table 1 also shows that no effect is seen from leisure time physical activity on HLDD. This indicates that strenuousness at work is the most important modifiable risk factor for hospitalization due to HLDD among men. Men in sedentary jobs may have HLDD not leading to hospitalization, but being treated as out-patients more often than men in strenuous jobs, a possibility which cannot be evaluated in this study.

### Methodological considerations

This study has the advantage of data from objectively measured physical fitness and an objectively evaluated low back disease. Furthermore, the study has the advantage of a prospective design among individuals without back disorders at baseline. In this study, physical fitness is given as aerobic capacity relative to body weight and therefore, naturally, fitness levels are highly associated with weight levels. The univariate analysis presents a high association between body weight and HLDD suggesting that weight might modify a possible association between high aerobic capacity and HLDD. Future studies should therefore investigate the role of directly measured aerobic capacity and the risk of HLDD and separately evaluate the possible modifying effect of body weight. Fitness varies considerably across age groups. For example, previously defined fitness categories suggest that a physical fitness of ~30 ml O_2_*kg^-1^*min^-1^ can be defined as “very poor” among males below the age of 30 and “very good” among males above the age of 75 [24]. In the current study, fitness above 32 ml O_2_*kg^-1^*min^-1^ has been defined as high fitness both for younger as well as for older males as the stratification of fitness in low and high categories in the current study was also statistically derived (i.e. based on the median value of the whole population). This may introduce a skewed distribution of age in the two fitness groups – i.e. many young males will fall in the category of high physical fitness and many older males will fall in the category of low physical fitness. However, since the age in this study ranged from 40–58 years, the median fitness of 32 ml O_2_*kg^-1^*min^-1^ only ranged from poor to fair according to previous categorisations. Thus, due to the relatively narrow age span, the skewness can be overcome by controlling for age in all analyses. The sufficiency of this action is supported by the fact that the current stratification of fitness previously has proven highly valid for the determination of other health outcomes [7,25]. However, the lack of repeated measures of physical fitness during the relatively long follow-up period may contribute to misclassification of physical fitness. One further limitation is the relatively small number of end-points, narrowing the possibility of conducting interaction analyses and increasing risk of a type-2 statistical error. Moreover, the inclusion of only middle-aged Caucasian men may make our results less relevant for other age groups, ethnic groups, and women.

## Conclusion

Among men without a history of low back pain or injury to the back, no association was found between the level of physical fitness and risk of hospitalisation due to HLDD during a 30-year follow-up. Strenuousness at work is observed to be the dominant modifiable risk factor for hospitalization due to HLDD, and initiatives at the workplace for reducing the strenuousness may be an important prevention method.

## Competing interests

The authors declare that they have no competing interests.

## Authors’ contributions

FG led the data collection. AH had the initial idea for the current study. PS led and conducted the statistical analyses. MBJ led the writing process. All authors contributed to the design of the study and the interpretation of data. All authors discussed the results and read and approved the final manuscript.

## Pre-publication history

The pre-publication history for this paper can be accessed here:

http://www.biomedcentral.com/1471-2474/14/86/prepub
